# Effects of dynamic resistance training on blood pressure across different baseline levels: a systematic review and meta-analysis

**DOI:** 10.3389/fcvm.2026.1843543

**Published:** 2026-05-29

**Authors:** ShengLong Luo, RuiQi Liu, Lijie Lou, JiaPeng Yang

**Affiliations:** 1 Faculty of Health Sciences and Physical Education, Macao Polytechnic University, Macao, Macao SAR, China; 2 School of Martial Arts and Traditional Chinese Sports, Tianjin University of Sport, Tianjin, China; 3 School of Martial Arts, Guangzhou Sport University, Guangzhou, China

**Keywords:** baseline blood pressure, blood pressure, dynamic resistance training, exercise prescription, hypertension, meta-analysis

## Abstract

**Objective:**

This study aimed to systematically evaluate the effects of dynamic resistance training (DRT) on populations with varying baseline blood pressure (BP) levels, compare the commonalities and differences across subgroups, and explore potential sources of heterogeneity.

**Methods:**

A systematic literature search of databases including PubMed, Embase, the Cochrane Library, and Web of Science was conducted from inception to January 2026. Randomized controlled trials (RCTs) investigating DRT as the intervention and resting BP as the outcome were included. A random-effects model was employed to calculate the pooled mean differences (MDs) and 95% confidence intervals (CIs). Subgroup analyses were performed based on baseline BP classifications and antihypertensive medication status, while meta-regression was used to assess the moderating effect of age. The Risk of Bias 2 (RoB 2) tool was used to assess the risk of bias, and the certainty of evidence was evaluated using the GRADE approach.

**Results:**

Eighteen studies were ultimately included in this meta-analysis. For systolic blood pressure (SBP), 18 studies comprising 27 effect sizes and 328 participants were pooled, revealing a significant reduction following DRT (MD = −7.57 mmHg; 95% CI: [−9.40, −5.74]; *p* < 0.001). For diastolic blood pressure (DBP), 17 studies comprising 26 effect sizes and 313 participants were analyzed, also demonstrating a significant reduction (MD = −4.73 mmHg; 95% CI: [−6.41, −3.05]; *p* < 0.001). Subgroup analyses revealed varying magnitudes of BP reduction among participants with different baseline BP levels. For SBP, the stage 2 hypertension subgroup exhibited the greatest reduction (MD = −9.33 mmHg), followed by the high-normal (MD = −6.17 mmHg) and normal BP subgroups (MD = −5.16 mmHg). For DBP, the stage 1 hypertension subgroup experienced the most significant benefit (MD = −8.82 mmHg). Furthermore, unmedicated participants showed a greater reduction in SBP (MD = −10.06 mmHg) compared to medicated individuals (MD = −6.46 mmHg), whereas DBP reductions were highly consistent between the two groups. Meta-regression analysis indicated that age had no significant moderating effect on the BP-lowering efficacy.

**Conclusions:**

DRT significantly lowers BP in adults, with the magnitude of reduction varying across different baseline levels, generally demonstrating a trend where higher baseline BP yields greater antihypertensive benefits. DRT can be prioritized as a non-pharmacological intervention for patients with stage 2 hypertension. However, given the current limited certainty of evidence and high inter-study heterogeneity, these findings warrant further verification through high-quality RCTs.

**Systematic Review Registration:**

https://www.crd.york.ac.uk/PROSPERO/myprospero, PROSPERO CRD420261332947.

## Introduction

Hypertension is a progressive cardiovascular syndrome caused by complex and interrelated etiologies (1). It is also one of the primary modifiable risk factors for cardiovascular disease (CVD) (2). Studies have shown that elevated blood pressure has become the leading global cause of death and disability-adjusted life years (DALYs) lost (3, 4). Numerous epidemiological studies indicate that hypertension is not only a risk factor but also a primary cause of various cardiac, cerebral, and vascular complications, such as stroke, coronary heart disease, heart failure, atrial fibrillation, peripheral arterial disease, cognitive impairment, and dementia (5–7). Hypertension accounts for 54% of stroke events and 45% of cardiovascular disease deaths, respectively (8). Consequently, the World Health Organization (WHO) has ranked hypertension as the third leading cause of death globally, responsible for one in every eight deaths (9).

Controlling BP has become a public health imperative. Current international guidelines for the management of hypertension (e.g., AHA/ACC and ESC/ESH guidelines) consistently recommend lifestyle interventions as first-line therapy or as an adjunct to pharmacological treatment (10, 11). Among various non-pharmacological interventions, regular physical activity has been proven to exert significant antihypertensive effects (12). Resistance training has gradually garnered extensive attention from clinicians and researchers as an effective alternative or complementary strategy. It can be broadly categorized into two subgroups: dynamic resistance training (DRT) and isometric resistance training (IRT) (13). In clinical practice, IRT interventions for hypertension primarily consist of isometric handgrip (IHG) training. Although US guidelines recommend IHG training for hypertension management (14), its application as an independent exercise therapy has limitations.The musculoskeletal adaptations of IHG training are largely confined to the small muscle groups being exercised, offering a limited impact on overall health. In contrast, DRT stimulates the entire musculature, promoting systemic musculoskeletal and metabolic benefits (15).

DRT also known as strength training, refers to a form of exercise involving alternating muscle contractions and relaxations against an external resistance (16). Previous studies have demonstrated that DRT can significantly reduce both SBP and DBP, with higher baseline BP levels yielding more pronounced antihypertensive effects (17).

Although existing meta-analyses have preliminarily confirmed the overall BP-lowering efficacy of DRT, they possess important methodological limitations that constrain their clinical applicability. Two landmark meta-analyses by Cornelissen (13) and MacDonald et al. (17) reported that DRT reduces SBP by approximately 3.5–5.7 mmHg and DBP by 3.2–5.2 mmHg. However, both analyses pooled heterogeneous populations—combining normotensive, high-normal, and hypertensive individuals—into a single overall estimate. This aggregation approach, while providing a general effect size, obscures critical between-group differences and fails to answer the clinically essential question of which patients derive the greatest benefit from DRT.

Substantial heterogeneity in the magnitude of BP reduction remains across different studies. This inconsistency in results may be largely attributed to differences in participant characteristics, among which baseline BP level is an often overlooked yet crucial moderating variable (18). According to the law of initial values in physiology, the magnitude of the autonomic nervous system's response to a stimulus depends on its initial functional level; thus, individuals with higher baseline BP are likely to achieve significantly greater BP reductions from interventions compared to normotensive and high-normal BP populations (19, 20). However, most previous systematic reviews have either pooled normotensive, high-normal BP, and hypertensive individuals for analysis, or focused solely on a single hypertensive cohort, lacking a systematic and quantitative comparison of the antihypertensive effects across populations with distinct baseline BP levels. This pooling effect may obscure the true efficacy of DRT for specific subgroups, thereby limiting the precise application of exercise prescriptions.

To address these gaps and provide more targeted exercise recommendations for populations with different baseline BP levels, this study aims to comprehensively evaluate the effects of DRT on SBP and DBP across distinct baseline BP categories through a systematic review and meta-analysis. We hypothesize that DRT efficacy varies systematically by baseline BP category, and that identifying these differential responses will provide robust evidence-based support for clinicians and exercise rehabilitation specialists in developing personalized and precise exercise intervention protocols for hypertension.

## Methods

### Literature search

This meta-analysis strictly adheres to the reporting guidelines for systematic reviews and meta-analyses (21) and has been registered on the PROSPERO website (ID: CRD420261332947). A systematic search was conducted in the PubMed, Embase, Cochrane Library, Web of Science, Medline, APA PsycNet, ProQuest, Springer, and Scopus databases to include all studies related to the research topic from the establishment of each database up to January 2026. The specific search strategy is shown in Supplementary Material Table S1. After initially determining the included studies, the reference lists of these studies and similar studies (searched from PubMed, Embase, and Web of Science) were supplemented to ensure the comprehensiveness and sufficiency of the included literature.

### Literature screening

A researcher used EndNote20 software to independently remove duplicates from the retrieved literature. The other two researchers conducted an initial screening by reading the titles and abstracts of the retrieved studies in accordance with pre-established inclusion and exclusion criteria. For studies that could not be excluded based solely on their titles and abstracts, full-text reviews were conducted to determine if they met the inclusion criteria. If disagreements arose during the screening process, a third researcher was invited to arbitrate.

### Inclusion and exclusion criteria for the study

The inclusion and exclusion criteria of this study were formulated strictly in accordance with the PICOS framework. Details are shown in Supplementary Table S2.

### Data extraction and transformation

Blood pressure is classified by level and grade into: normal blood pressure (systolic blood pressure <120 mmHg and diastolic blood pressure <80 mmHg), high-normal blood pressure (systolic blood pressure 120–139 mmHg and/or diastolic blood pressure 80–89 mmHg), and hypertension (systolic blood pressure ≥ 140 mmHg and/or diastolic blood pressure ≥ 90 mmHg) (22–24).Two researchers independently extracted data, including literature sources and author details, basic descriptive information of the included studies, demographic characteristics of the study participants, specific protocols of dynamic resistance training interventions, and measurement results of blood pressure indicators before and after the intervention. All extracted data were reviewed by a third researcher. Any disagreements were submitted to a fourth researcher for final and binding arbitration. If data in a study were missing or presented only in graphical form, an attempt was first made to contact the original authors to obtain the required data. If contact was unsuccessful, GetData Graph Digitizer 2.25 software was used to extract data presented only in graphical form. If the relevant data could not be obtained eventually, the study was excluded from this meta-analysis. For data reported as standard error (SE) or confidence intervals (CIs), they were converted to standard deviation (SD) using the formula recommended in the Cochrane Handbook (25). Finally, the resting systolic blood pressure and resting diastolic blood pressure values in the awake state before and after the intervention (expressed as mean ± standard deviation) were selected as the primary outcome indicators.

### Risk of bias assessment

Two researchers independently assessed the risk of bias in the included studies using the Risk of Bias Assessment Tool 2 (RoB 2) recommended by Cochrane, covering aspects such as the randomization process, deviations from the intended intervention, missing outcome data, outcome measurement, and selection of reported results (26).

### Quality of evidence

The certainty of evidence was assessed using the Grading of Recommendations Assessment, Development and Evaluation (GRADE) system (27). Two reviewers independently evaluated the quality of the evidence, and any disagreements were resolved through discussion or consultation with a third reviewer. In the GRADE system, evidence from randomized controlled trials is initially rated as “high” quality. During the assessment process, the quality of evidence is downgraded (by 1 or 2 levels) based on the severity of five downgrading factors: risk of bias, inconsistency, indirectness, imprecision, and presence of publication bias. It is upgraded based on three upgrading factors: large effect size, presence of a dose-effect relationship, and all reasonable residual confounding factors that would reduce the evaluated effect (28). The final quality of evidence is determined into four grades: “high”, “moderate”, “low”, or “very low”.

### Definition of baseline blood pressure Strata

Baseline BP levels of participants were pre-specified and stratified into four mutually exclusive categories in accordance with the 2017 ACC/AHA Guideline for the Prevention, Detection, Evaluation, and Management of High Blood Pressure in Adults (11), with reference to the 2021 WHO hypertension treatment guideline (22) and 2024 ESC/ESH hypertension guidelines (23). The specific thresholds for each stratum are as follows:

Normal BP: SBP <120 mmHg and DBP <80 mmHg;

High-normal BP (Prehypertension): SBP 120–139 mmHg and/or DBP 80–89 mmHg;

Stage 1 Hypertension: SBP 140–159 mmHg and/or DBP 90–99 mmHg;

Stage 2 Hypertension: SB*P* ≥ 160 mmHg and/or DB*P* ≥ 100 mmHg.

Participants with hypotension (defined as SBP <90 mmHg and/or DBP <60 mmHg) were excluded from this meta-analysis according to the pre-established exclusion criteria, and no relevant subgroup analysis was performed for this population.

### Statistical analysis

In this meta-analysis, data analysis was performed using RStudio software with the “meta”, “metafor”, and “tidyverse”packages (29). The inverse variance method based on the Der Simonian-Laird approach was employed to pool the primary effects through a random-effects model. The Jackson method was applied to calculate tau^2^, tau, and their corresponding 95% confidence intervals (95%CI) (30). The mean difference (MD) and its 95%CI were selected as the effect size indicators. The statistics for effect analysis included the pooled MD under the random-effects model, 95%CI, t-statistic, and the corresponding *p*-value (9). The following indicators were used to comprehensively assess heterogeneity: the Cochrane Q test and its *p*-value were used to determine the statistical significance of heterogeneity; the I^2^ statistic and prediction interval were used to quantify the degree of heterogeneity (31). The degree of heterogeneity was classified according to the I^2^ statistic: 0%–30% as mild heterogeneity, 31%–50% as moderate heterogeneity, 51%–75% as high heterogeneity, and 76%–100% as very high heterogeneity (32). Subgroup analysis and meta-regression analysis were conducted to explore the sources and influencing factors of heterogeneity. The Bonferroni correction method was used for subgroup analysis to adjust the significance threshold (33). A sensitivity analysis was performed using the one-by-one exclusion method to evaluate the robustness of the pooled primary effects. A funnel plot was used to investigate publication bias (34), supplemented by the Egger test (35). The significance level for the main effects of the full model was set at *α* = 0.05, while the significance level for subgroup analysis was strictly Bonferroni-adjusted according to the number of comparisons (i.e., *α*’ = 0.05/number of comparisons).

## Result

### Study selection

**Figure 1:**
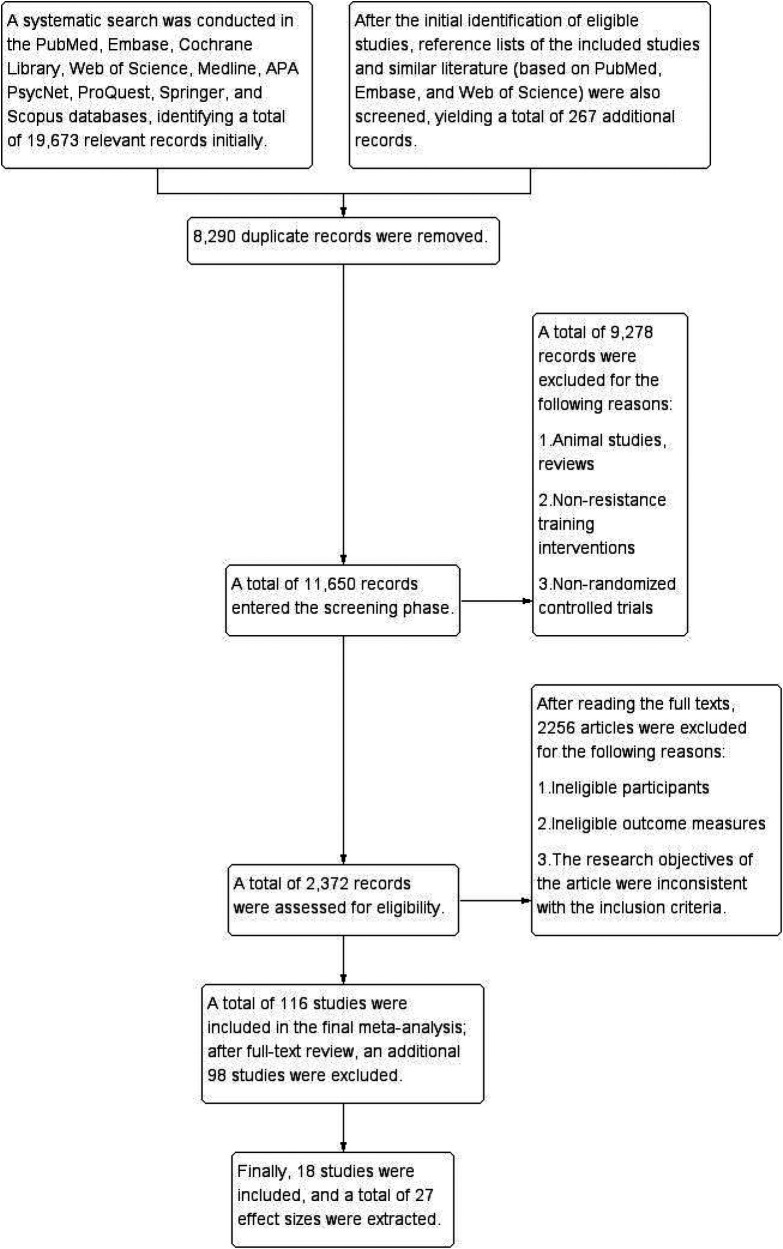
Literature screening process.

The initial search identified a total of 19,673 records across all databases. After applying the inclusion and exclusion criteria, 18 studies were ultimately included in the meta-analysis. The detailed study selection process is illustrated in Figure 1.

### Characteristics of included studies

A total of 18 studies (yielding 27 effect sizes) were included in this meta-analysis. Published between 2013 and 2024, these studies aimed to investigate the effects of dynamic resistance training on populations with varying baseline resting blood pressure levels. The included studies involved a total of 328 participants aged between 44 and 72 years, with sample sizes ranging from 5 to 22 participants per study. Additional details are provided in Supplementary Figure S1.

### Meta-analysis results

**Figure 2:**
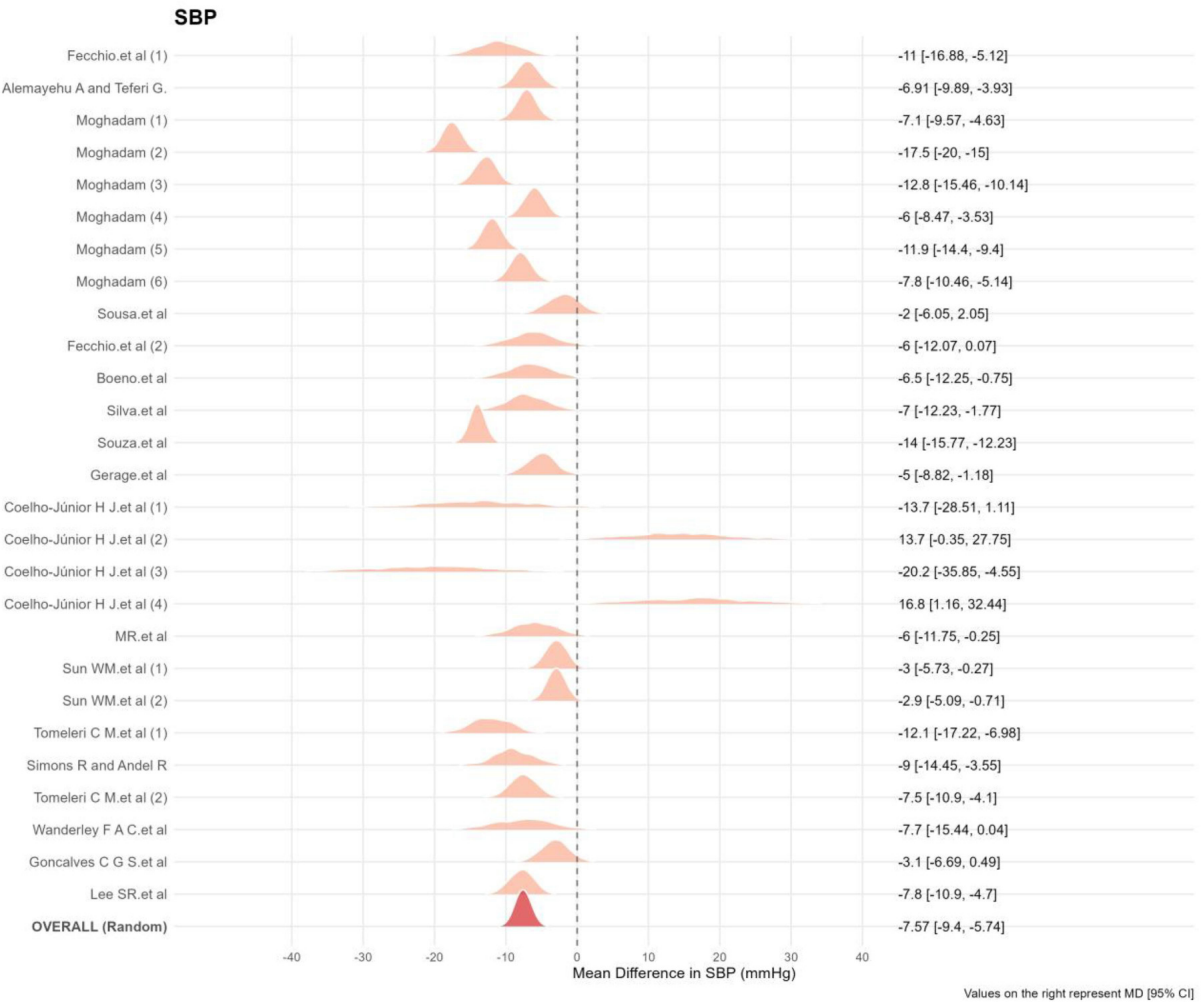
Ridge plot of meta-analysis on the effect of dynamic resistance training on systolic blood pressure. Left: included individual studies; Right: corresponding study effect sizes (MD) and 95% confidence intervals. Orange areas: effect size distribution and weight proportion of individual studies; red area at bottom: pooled overall effect size from the random-effects model.

**Figure 3:**
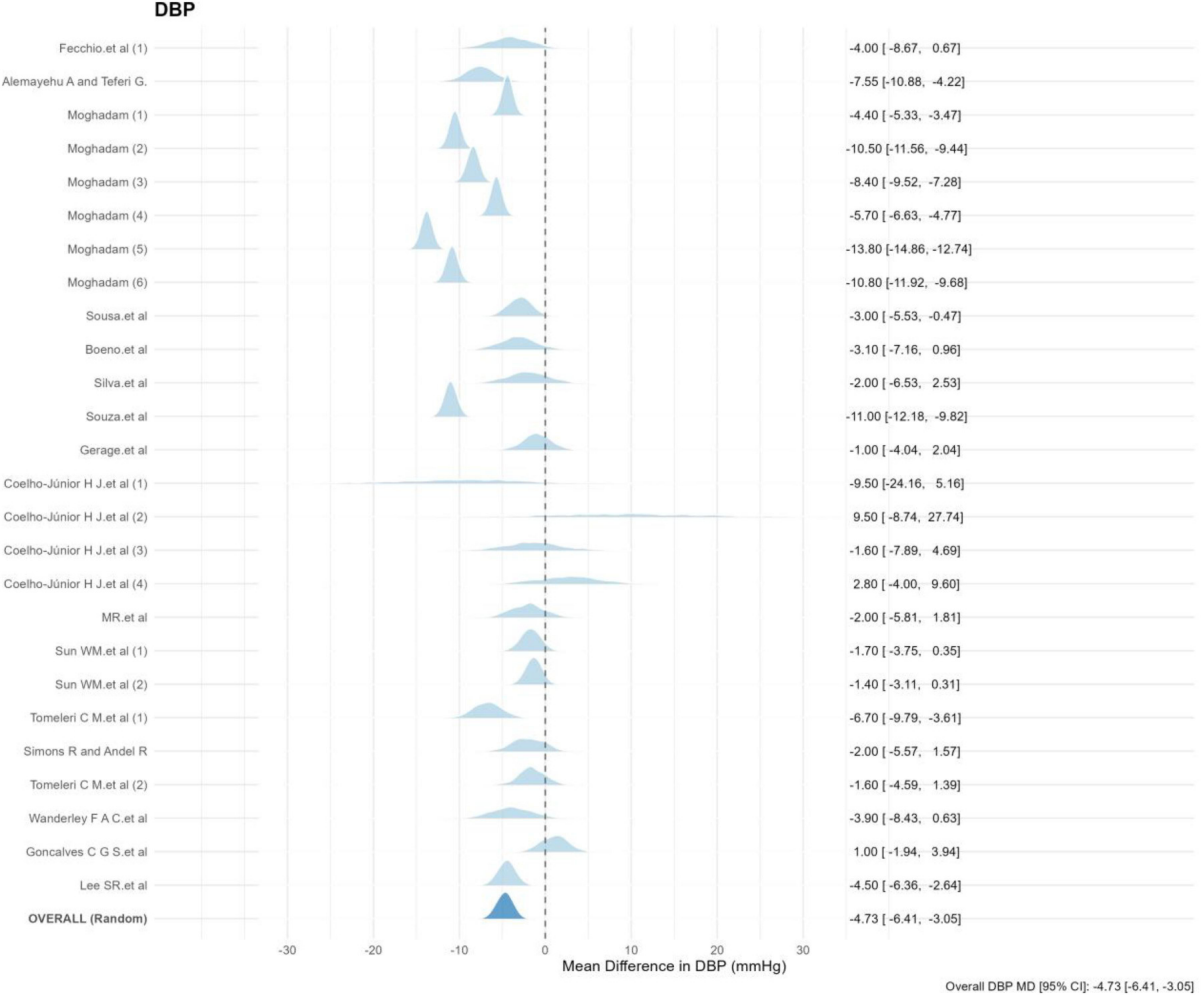
Ridge plot of meta-analysis on the effect of dynamic resistance training on diastolic blood pressure. Left: included individual studies; Right: corresponding study effect sizes (MD) and 95% confidence intervals. Light blue areas: effect size distribution and weight proportion of individual studies; dark blue area at bottom: pooled overall effect size from the random-effects model.

The meta-analysis demonstrated that DRT significantly reduced blood pressure in adults compared to pre-training levels (Figures 2, 3). The pooled MD for SBP was −7.57 mmHg (95% CI: −9.40 to −5.74; *p* < 0.001), and the pooled MD for DBP was −4.73 mmHg (95% CI: −6.41 to −3.05; *p* < 0.001). However, significant inter-study heterogeneity was detected for both outcomes (SBP: I^2^ = 87.0%, Cochran's *Q* test *p* < 0.001; DBP: I^2^ = 95.1%, *p* < 0.001). To elucidate the potential sources of this heterogeneity, subgroup and meta-regression analyses were conducted.

### Subgroup analyses

**Figure 4:**
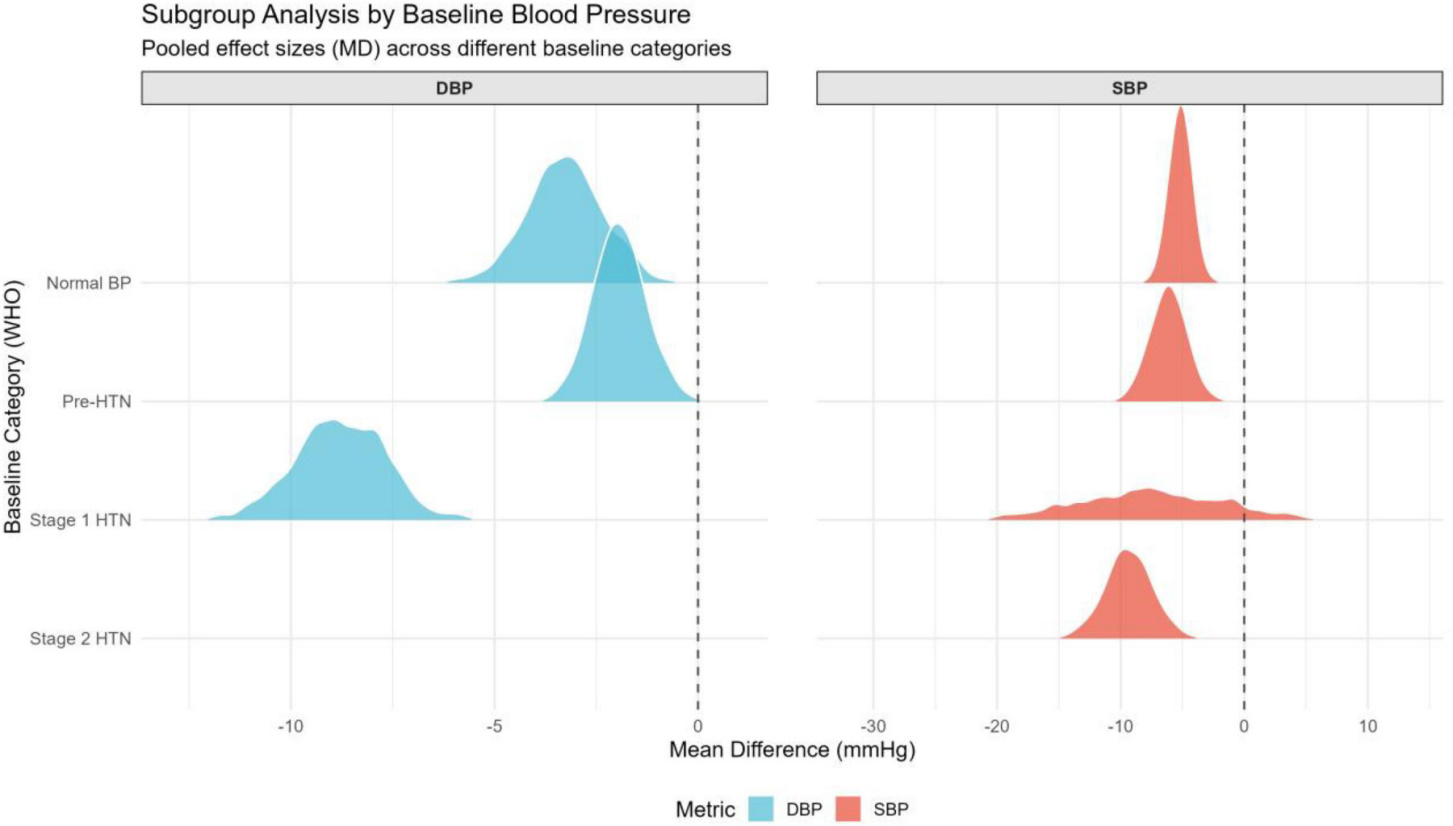
Subgroup analysis of the antihypertensive effect of dynamic resistance training intervention on different baseline blood pressure levels. The peak shape in the figure represents the distribution of the combined effect size of the corresponding subgroup. The center position of the peak represents the combined effect size of the subgroup, and the width of the peak represents the degree of variation in the effect size (95% confidence interval range).

**Figure 5:**
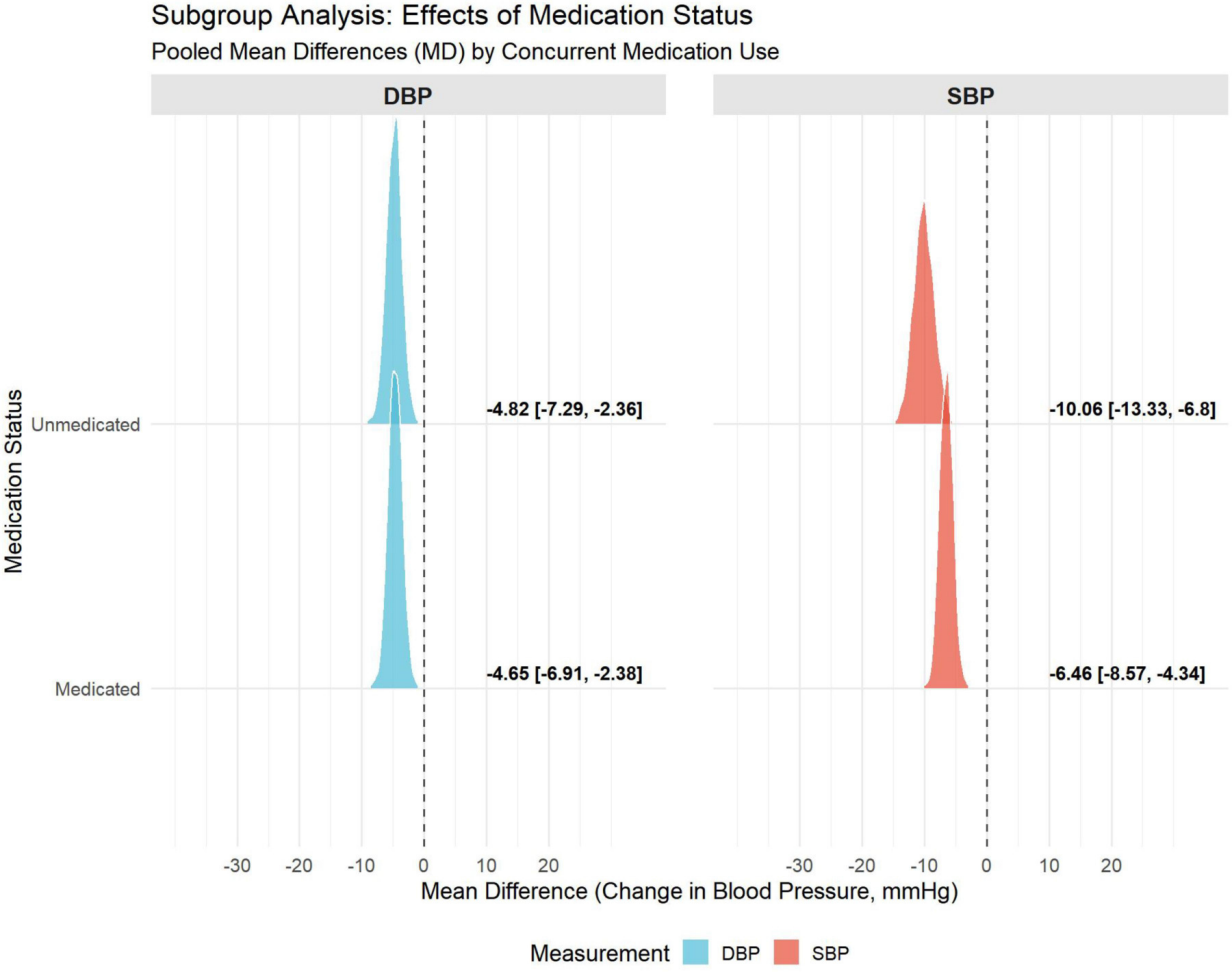
Subgroup analysis of the antihypertensive effect of interventions stratified by baseline combined medication status. The peak shape in the figure represents the distribution of the combined effect size of the corresponding subgroup, and the values marked next to the peak are the combined effect size (MD) and 95% confidence interval of the corresponding subgroup.

Subgroup analysis based on baseline BP revealed that the antihypertensive effects of DRT varied significantly according to the participants' baseline BP classifications (Figure 4). Regarding SBP, the stage 2 hypertension subgroup exhibited the greatest reduction (MD = −9.33 mmHg; 95% CI: −13.10 to −5.55), followed by the prehypertension subgroup (MD = −6.17 mmHg) and the normotensive subgroup (MD = −5.16 mmHg), all of which were statistically significant. In contrast, although the pooled point estimate for SBP in the stage 1 hypertension subgroup showed a downward trend (MD = −7.54 mmHg), it did not reach statistical significance (95% CI: −19.40 to 4.32; I^2^ = 80.0%).DBP exhibited a different reduction pattern. The most pronounced DBP reduction was observed in participants with stage 1 hypertension (MD = −8.82 mmHg; 95% CI: −11.00 to −6.64), whereas the reductions in normotensive and high-normal participants were relatively smaller but remained statistically significant (MD = −3.25 mmHg and −1.93 mmHg, respectively).Subgroup analysis based on the concurrent use of antihypertensive medication is presented in Figure 5. For SBP, both the unmedicated group (MD = −10.06 mmHg) and the medicated group (MD = −6.46 mmHg) achieved statistically significant antihypertensive benefits (evaluated using a Bonferroni-corrected 97.5% CI). Although the unmedicated group demonstrated a numerically larger reduction trend, the difference in BP reduction between the two groups did not reach statistical significance following rigorous correction for multiple comparisons (adjusted *p* = 0.139). However, for DBP, the antihypertensive effects were highly consistent between the unmedicated group (MD = −4.82 mmHg; 95% CI: −7.29 to −2.36) and the medicated group (MD = −4.65 mmHg; 95% CI: −6.91 to −2.38).

### Meta-Regression analysis

**Figure 6:**
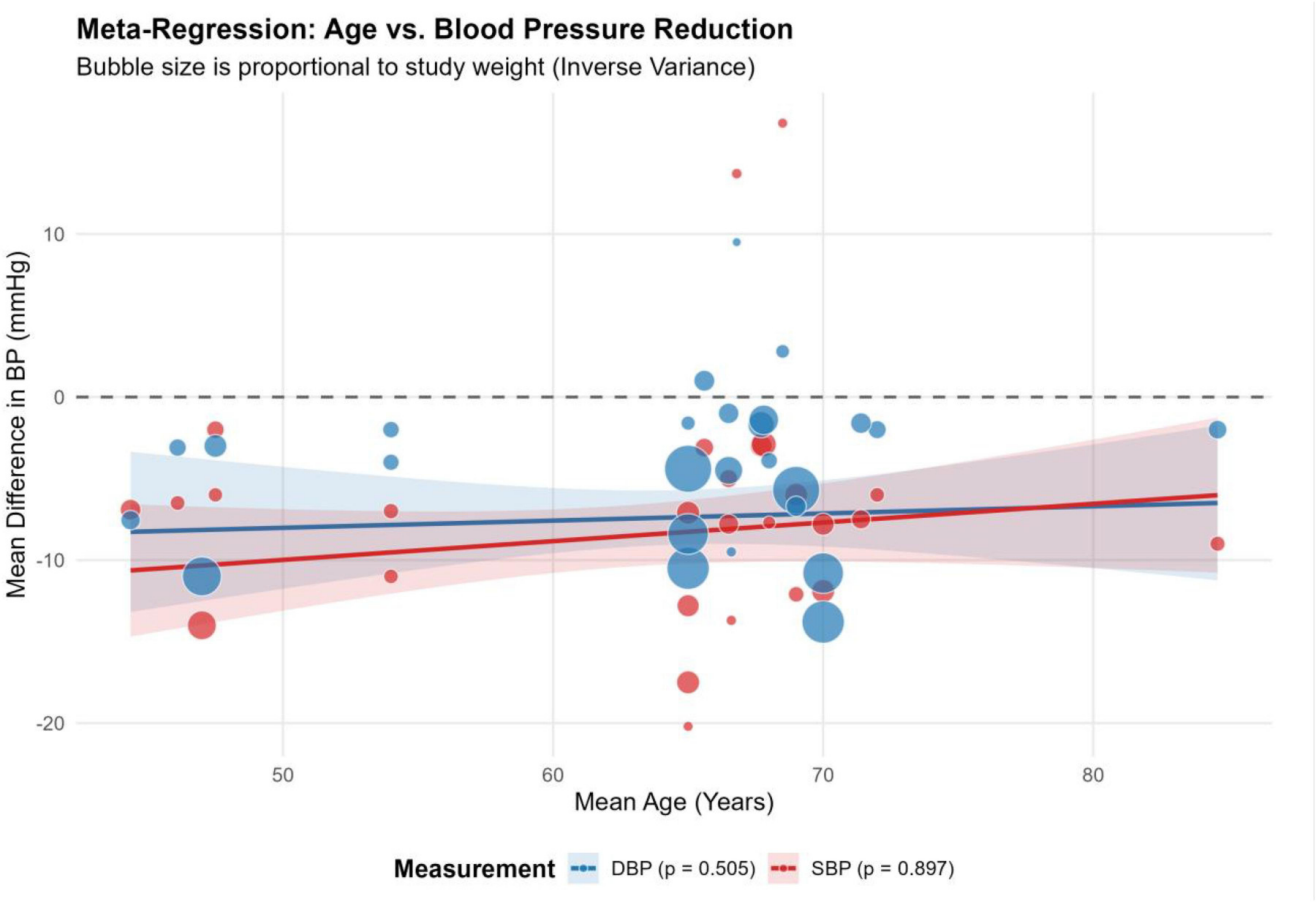
Bubble plot of regression analysis on the association between the average age of subjects and the antihypertensive effect of intervention. Bubbles represent included studies, with size proportional to study weight calculated by the inverse variance method. Blue bubbles/regression line: DBP; red bubbles/regression line: SBP. Solid lines: meta-regression fitted lines; translucent bands: 95% confidence intervals of regression estimates.

**Figure 7:**
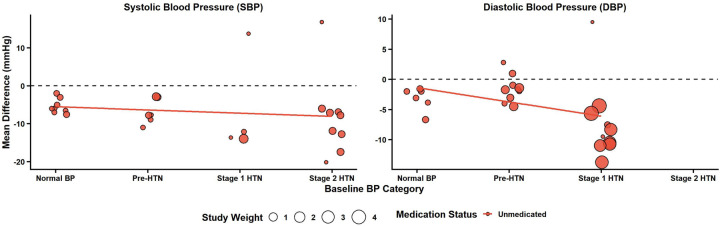
Scatter bubble chart of the antihypertensive effect of intervention in untreated populations under different baseline blood pressure classifications. Bubble plot of intervention effects stratified by baseline BP status. Left panel: SBP effect distribution; Right panel: DBP effect distribution. *X*-axis: WHO baseline BP categories (normal, high-normal, grade 1 HTN, grade 2 HTN); *Y*-axis: BP change MD (mmHg). Horizontal dashed line: null effect line (MD = 0); lower values indicate greater BP reduction. Bubbles: included studies; bubble size proportional to Meta-analysis weight (legend at bottom). Red solid line: fitted trend line for non-medicated populations.

A random-effects meta-regression analysis, estimated using restricted maximum likelihood (REML), was employed to evaluate the potential moderating effect of participants' age as a continuous covariate on the intervention effect (Figure 6). The results indicated that the mean age of the participants did not significantly predict the intervention effect for either SBP (*β* = 0.0126, *p* = 0.897) or DBP (*β* = 0.0612, *p* = 0.505). These findings suggest that within the age range covered by the included studies, the antihypertensive benefits of DRT are consistent across age groups and are not significantly moderated by participant age.To further explore the potential synergistic impact between baseline BP and medication status, an interaction meta-regression analysis was conducted using a mixed-effects model.The results revealed that the overall regression model was statistically significant (QM = 9.58, *p* = 0.0225) and accounted for 27.41% of the inter-study heterogeneity (R^2^ = 27.41%). Crucially, the analysis indicated no significant interaction between baseline BP category and medication status (*β* = 1.61, *p* = 0.255). After controlling for the interaction term, the baseline BP category still demonstrated a significant independent moderating effect (*β* = −2.46, *p* = 0.032) (Figure 7).

### Risk of bias assessment

**Figure 8:**
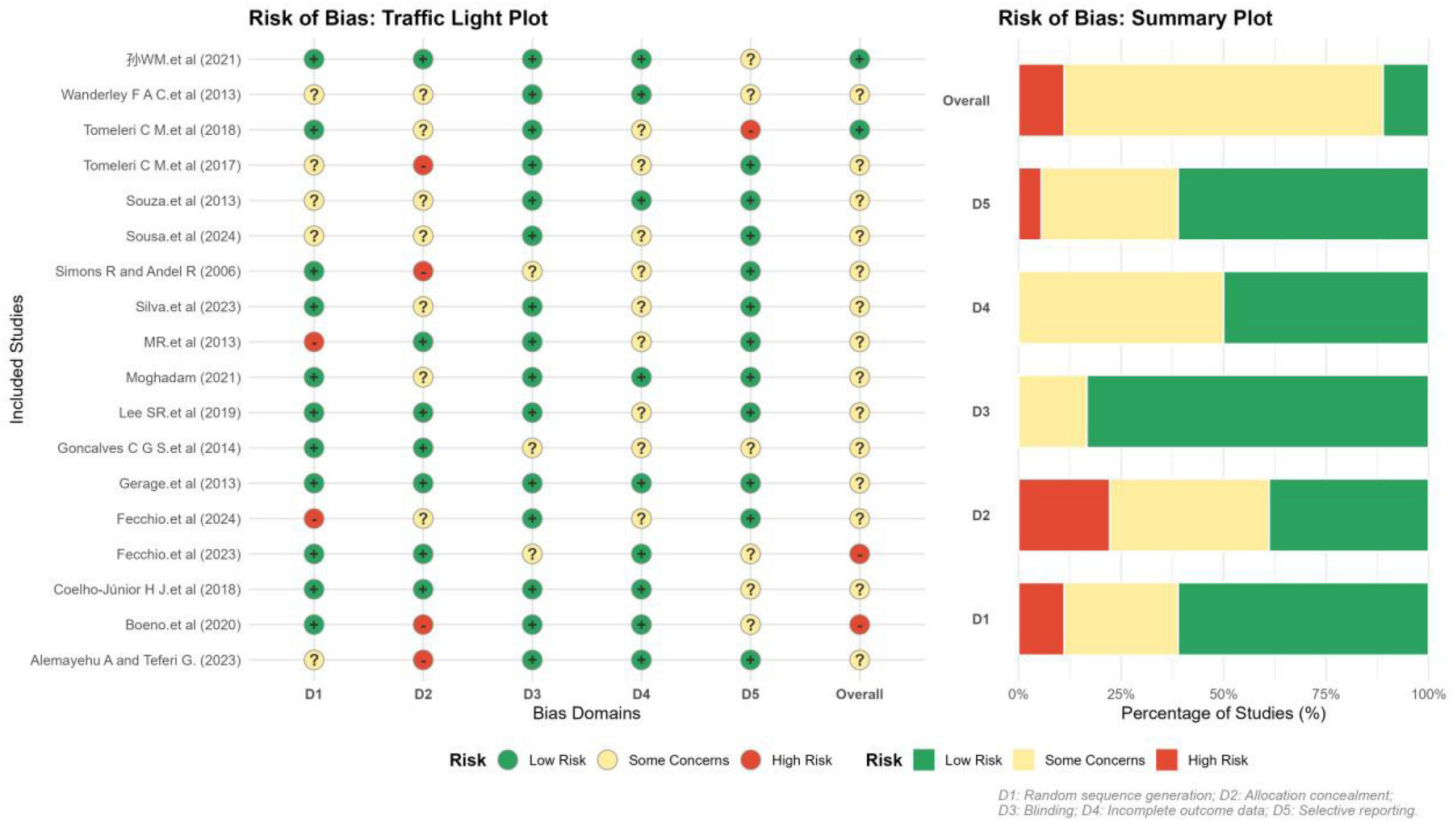
Risk of bias assessment chart of cochrane included in the study. This figure is drawn based on the Cochrane RoB 2.0 risk of bias assessment tool, consisting of a traffic light chart on the left and a summary chart on the right, comprehensively displaying the risk of bias of the included studies.

Overall, the methodological quality of the included studies varied. The majority of the studies were assessed as having “some concerns” regarding the risk of bias, with relatively few providing high-quality (low risk of bias) evidence (Figure 8).

Low Risk of Bias: Two studies (11.1%), Sun W.M. et al. and Tomeleri C.M. et al., were categorized as having a low risk of bias. These studies demonstrated high methodological rigor in random sequence generation, data integrity, and selective reporting, thereby providing highly credible data on the antihypertensive effects.

Some Concerns: Fourteen studies (77.8%), comprising the vast majority of the included literature (e.g., Moghadam, Fecchio et al., Sousa et al.), were rated as having some concerns. This is a common occurrence in exercise intervention trials, primarily because studies often fail to report specific details regarding allocation concealment. Additionally, the inherent nature of exercise interventions makes it challenging to implement strict blinding for participants and personnel, thereby introducing potential methodological blind spots (61).

High Risk of Bias: Two studies (11.1%), Fecchio et al. and Boeno et al., were deemed to have a high risk of bias. This suggests the presence of distinct methodological flaws in the design or execution of these trials that could compromise the validity of the intervention outcomes.

### Sensitivity analysis

**Figure 9:**
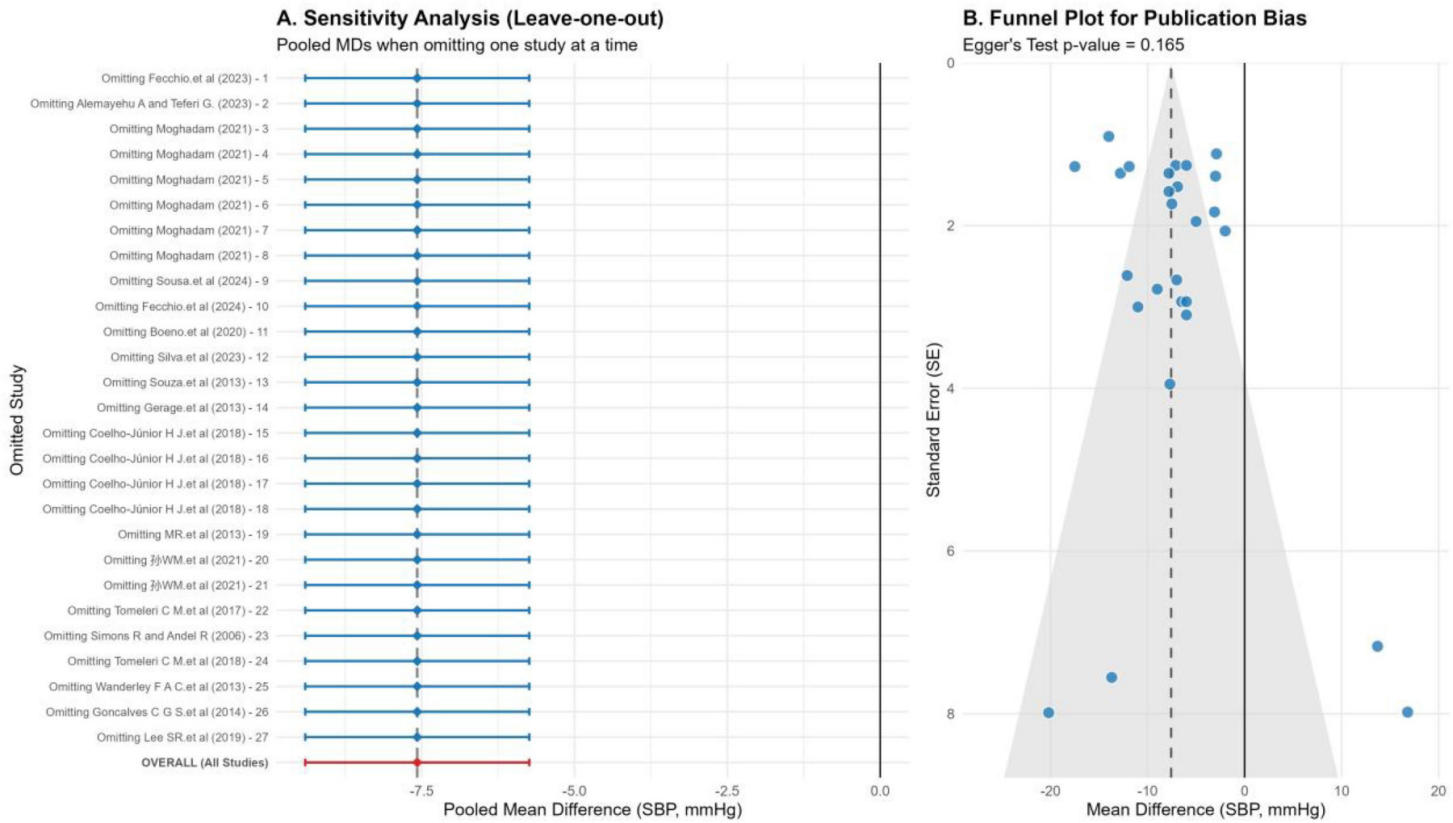
Sensitivity analysis and publication bias test chart of systolic blood pressure meta-analysis. **(A)** Leave-one-out sensitivity analysis. *Y*-axis: excluded studies; *X*-axis: SBP MD and 95% CI of remaining studies; red horizontal line: overall pooled effect size. **(B)** Funnel plot for publication bias assessment. *X*-axis: SBP change MD; *Y*-axis: standard error of effect size; vertical dashed line: overall pooled effect size; solid lines: 95% CI boundaries; scatter points: included studies.

**Figure 10:**
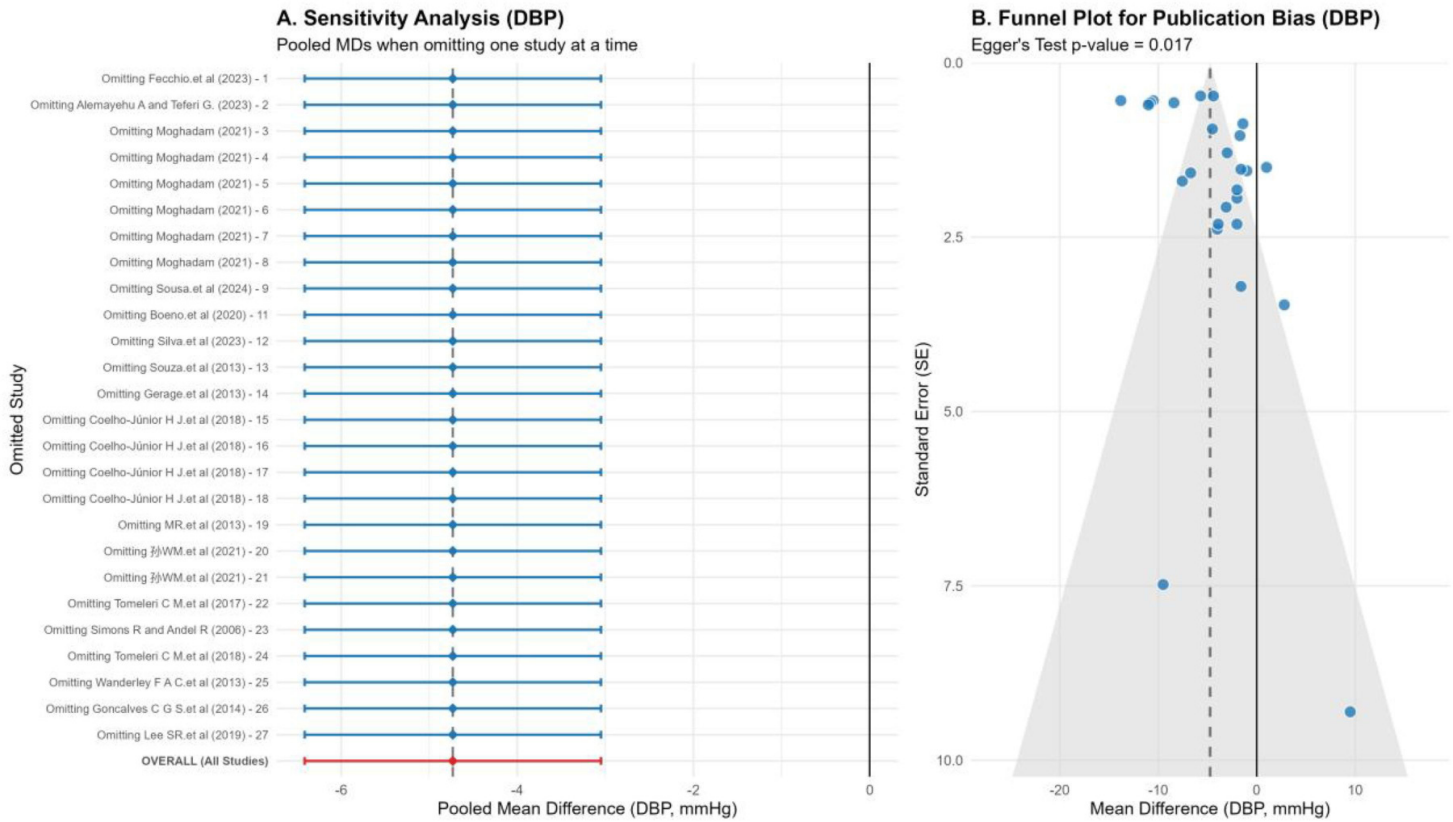
Sensitivity analysis and publication bias test chart of diastolic blood pressure meta-analysis. **(A)** Leave-one-out sensitivity analysis. *Y*-axis: excluded studies; *X*-axis: DBP MD and 95% CI of remaining studies; red horizontal line: overall pooled effect size. **(B)** Funnel plot for publication bias assessment. *X*-axis: DBP change MD; *Y*-axis: SE of effect size; vertical dashed line: overall pooled effect size; solid lines: 95% CI boundaries; scatter points: included studies.

To evaluate the robustness of the overall pooled effects, a sensitivity analysis was conducted using the leave-one-out method. The results demonstrated that for both SBP and DBP, the recalculated pooled MDs and their 95% CIs did not substantially change after iteratively omitting any single included study, and they consistently remained highly statistically significant (all *p* < 0.001). This indicates that the primary findings of this meta-analysis are highly robust, and the overall antihypertensive effect of the intervention was not driven by any single extreme study or outlier (Figures 9, 10).

### Publication bias

Potential publication bias and small-study effects were systematically evaluated through visual inspection of funnel plot symmetry, in conjunction with Egger's regression test. For SBP, the funnel plot displayed a relatively symmetrical distribution, and Egger' s test confirmed the absence of significant publication bias (*p* = 0.165).However, the analysis for DBP revealed a degree of asymmetry in the funnel plot, and Egger' s test reached statistical significance (*p* = 0.017). This statistical outcome suggests the potential presence of publication bias or a small-study effect in the pooled DBP data (i.e., smaller studies showing minor or non-significant effects might remain unpublished) (Figures 9, 10). A trim-and-fill iterative analysis estimated 11 potentially missing studies, which were imputed into the funnel plot to restore symmetry. After including these imputed studies, the pooled effect size for DBP was re-estimated. The results showed that, following correction, the pooled MD for DBP remained highly significant at −7.83 mmHg (95% CI: −9.88 to −5.79, *p* < 0.0001).

### Certainty of evidence

Regarding the SBP-lowering effect, although the overall pooled effect was highly significant, the certainty of evidence was downgraded to “low”.This decision was driven by the prevalent methodological concerns among the included studies (nearly 80% had “some concerns”, representing a serious risk of bias) and the extremely high inter-study heterogeneity (I^2^ = 87.0%, indicating serious inconsistency).For the DBP-lowering effect, in addition to facing the same serious risk of bias and inconsistency (I^2^ = 95.1%) as SBP, Egger's test also revealed potential publication bias (*p* = 0.017). Consequently, the certainty of evidence for DBP was further downgraded to “very low”. The certainty of evidence for each outcome was evaluated using the GRADE framework, and the detailed assessment is presented in Supplementary Table S3.

## Discussion

The primary objective of this study was to elucidate the effects of DRT on populations with varying baseline resting BP levels and to explore potential moderating factors. Our findings suggest that DRT may reduce resting BP in adult populations, with reductions of 7.57 mmHg in SBP and 4.73 mmHg in DBP. If confirmed in higher-quality studies, this magnitude of reduction could carry clinical significance, as previous epidemiological evidence indicates that a 5 mmHg reduction in SBP is associated with approximately 13% decreased risk of stroke and 10% decreased risk of coronary heart disease (36), while a 10 mmHg reduction in SBP correlates with an estimated 20% reduction in overall cardiovascular event risk (37). However, given the low-to-very-low certainty of current evidence (GRADE assessment) and very high heterogeneity (I^2^ = 87%–95%), these estimates should be interpreted cautiously.

Furthermore, through systematic subgroup analyses, this study reveals a differentiated pattern of antihypertensive effects across distinct baseline BP categories. By directly and quantitatively comparing four subgroups—normal BP, high-normal BP, stage 1 hypertension, and stage 2 hypertension—within the same meta-analytical framework, this study provides evidence that may support more targeted exercise prescription formulation.

Our findings both corroborate and extend previous meta-analytic evidence, while addressing critical methodological limitations of earlier work. Cornelissen and Fagard (13) reported that DRT reduced resting SBP by approximately 3.5 mmHg and DBP by 3.2 mmHg, while MacDonald et al. (17) found comparable reductions (SBP: −5.7 mmHg; DBP: −5.2 mmHg). The somewhat larger SBP reduction observed in our study (−7.57 mmHg) likely reflects three key methodological differences:First, our study included a higher proportion of hypertensive patients (incorporating both stage 1 and stage 2 subgroups), and existing literature suggests (38) that individuals with higher baseline BP may exhibit greater responsiveness to exercise interventions. Second, only DRT delivered as an isolated intervention was included in the present review, while studies investigating mixed exercise interventions (such as concurrent aerobic and resistance training) were explicitly excluded. This design permitted a more precise evaluation of the independent antihypertensive efficacy of DRT.Third, By stratifying participants into four distinct BP categories and analyzing them separately, we revealed that the magnitude of BP reduction varies systematically by baseline BP level—an insight obscured in previous pooled analyses. This stratified approach provides clinically actionable information that generic pooled estimates cannot: it identifies which patient subgroups may derive the greatest benefit and quantifies the expected magnitude of benefit for each category. This represents a step toward more precision-oriented exercise prescription, moving beyond generic “one-size-fits-all” recommendations.

Recently, Edwards et al. (39) reported an SBP reduction of approximately 8.2 mmHg with IRT. However, as noted in the introduction, IHG training primarily targets isolated, small muscle groups, offering limited systemic musculoskeletal and metabolic benefits (40). In contrast, DRT not only appears to achieve comparable antihypertensive effects but may concurrently promote improvements in muscle strength, bone mineral density, metabolic function, and body composition (15, 41, 62), potentially offering broader health advantages.

One of the most important findings is that DRT's antihypertensive effect appears to be moderated by baseline BP levels, with SBP and DBP demonstrating distinct patterns. Regarding SBP, the stage 2 hypertension subgroup exhibited the greatest reduction (MD = −9.33 mmHg), followed by the high-normal BP (MD = −6.17 mmHg) and normal BP subgroups (MD = −5.16 mmHg). This finding is consistent with previous research (38) and aligns with the law of initial values, a physiological principle suggesting that individuals with higher baseline dysfunction may have greater capacity for improvement (42).

Pathophysiologically, hypertensive patients often present with sympathetic nervous system overactivation, vascular endothelial dysfunction, and increased arterial stiffness (42), potentially providing a larger therapeutic window for exercise interventions. DRT may exert antihypertensive effects in hypertensive populations through multiple potential mechanisms, including possibly dampening sympathetic nerve activity (43), enhancing endothelium-dependent vasodilation (44), and reducing peripheral vascular resistance (45).

Importantly, the SBP reduction in the stage 1 hypertension subgroup did not reach statistical significance (95% CI crossed zero). However, this should not be interpreted as evidence of DRT ineffectiveness for this population. The pooled point estimate (MD = −7.54 mmHg) indicates a clinically substantial reduction comparable to the stage 2 hypertension subgroup (MD = −9.33 mmHg). *post-hoc* power analysis revealed that, given only 4 included studies and high inter-study heterogeneity (I^2^ = 80.0%), statistical power was merely ∼24% (well below the conventional 80% threshold). This strongly suggests the non-significant result likely represents a Type II error (false negative) driven by insufficient sample size (46) rather than true lack of efficacy. Large-scale, rigorously designed RCTs specifically targeting stage 1 hypertension are urgently needed to provide more precise effect estimates.

Regarding DBP, the reduction pattern differed markedly from SBP. The stage 1 hypertension subgroup experienced the most pronounced DBP reduction (MD = −8.82 mmHg), far exceeding normotensive (MD = −3.25 mmHg) and high-normal BP subgroups (MD = −1.93 mmHg). This discordance between SBP and DBP responses likely reflects their distinct pathophysiological determinants: SBP is predominantly influenced by large artery stiffness and cardiac output, whereas DBP depends more heavily on peripheral vascular resistance and small vessel function (47). DRT's potential regulatory effects on sympathetic vasoconstrictor activity may yield particularly pronounced improvements in the elevated peripheral resistance characteristic of stage 1 hypertension (48), possibly explaining the prominent DBP benefits in this subgroup.

Concurrent antihypertensive medication appeared to differentially moderate SBP but not DBP responses. Unmedicated participants achieved numerically larger SBP reductions (MD = −10.06 mmHg) compared to medicated individuals (MD = −6.46 mmHg). This may be because medicated patients already maintain lower BP levels through pharmacotherapy, potentially compressing the physiological headroom for further SBP reductions (49). Additionally, certain drug classes (e.g., beta-blockers) may blunt exercise-induced cardiovascular responses (50), and medicated patients often have longer disease duration and more severe vascular remodeling, potentially attenuating exercise efficacy (51).

In contrast, DBP reductions were highly consistent between groups (unmedicated: −4.82 mmHg vs. medicated: −4.65 mmHg), suggesting DRT may lower DBP through pathways relatively independent of and complementary to pharmacological mechanisms. Given that DBP primarily reflects peripheral vascular resistance, DRT may reduce this resistance by potentially improving endothelial function, promoting nitric oxide bioavailability (52), and modulating sympathetic tone (53, 63). This finding suggests DRT could serve as an effective adjunct to pharmacotherapy, providing additional BP reductions even in medicated patients, particularly for DBP.

The interaction between medication status and baseline BP did not reach statistical significance (*P* = 0.255), indicating insufficient evidence within the current dataset to demonstrate that medication substantially modifies the graded association between baseline BP and antihypertensive benefits. However, given sample size imbalances between medicated and unmedicated subgroups, statistical power to detect such interactions may be limited. The incremental BP reduction across baseline categories (SBP decreasing by an additional 2.46 mmHg per escalating BP tier) suggests that patients with more severe hypertension, despite typically having more compromised endothelial function, may paradoxically possess greater physiological potential for improvement through resistance training.

While evidence for the potential antihypertensive benefits of DRT has been provided by this meta-analysis, consideration of optimal exercise prescription parameters is required for the translation of these findings into clinical practice.Based on the included studies, we observed the following characteristics across interventions: Most studies employed moderate-to-high intensity training (60%–80% of 1RM), though some used lower intensities (40%–60% 1RM) with higher repetitions. Current evidence does not definitively establish the optimal intensity for BP reduction.The majority prescribed 2–3 sessions per week, consistent with general resistance training guidelines. Some employed higher frequencies (4–5 sessions/week), but whether increased frequency enhances antihypertensive effects remains unclear.Intervention periods ranged from 6 to 24 weeks. Most studies observed significant BP reductions within 8–12 weeks, suggesting this may represent a reasonable minimum duration. However, longer-term studies (≥6 months) are scarce.Protocols predominantly included multi-joint compound exercises (e.g., leg press, chest press, lat pulldown) targeting major muscle groups. Whole-body resistance training may be preferable to isolated muscle group training for maximizing systemic hemodynamic adaptations. Common prescriptions included 2–4 sets of 8–12 repetitions per exercise, though considerable variation existed. The very high heterogeneity in training protocols (I^2^ = 87%–95%) prevented robust subgroup analyses to identify optimal prescription parameters. This represents a critical evidence gap. Future factorial design RCTs systematically comparing different intensities, volumes, and frequencies are essential to establish evidence-based prescription guidelines.

The very high heterogeneity observed (I^2^ = 87% for SBP; 95% for DBP) substantially limits confidence in the pooled estimates and warrants careful interpretation. Beyond baseline BP and medication status, several additional factors likely contribute:1. Training Protocol Variability: DRT prescriptions varied drastically in intensity (40%–80% 1RM), frequency (2–5 sessions/week), duration (6–24 weeks), exercise selection, volume, and rest intervals. Each parameter may independently influence antihypertensive responses, creating complex, multifactorial heterogeneity.2. BP Measurement Methodology: Studies employed inconsistent assessment methods—from office measurements to automated monitors, with variations in timing, posture, number of readings, and averaging procedures. Office measurements are susceptible to white-coat effects, potentially inflating baseline values and overestimating efficacy (54).3.Participant Characteristics:Inadequate reporting precluded systematic exploration of additional moderators including sex distribution, body mass index, race/ethnicity, comorbidity burden, baseline fitness level, and dietary patterns—all of which may modify exercise responses.4. Study Quality and Design: Methodological heterogeneity (risk of bias variations, blinding procedures, adherence monitoring) may introduce performance and detection bias.5. Small-Study Effects: Many studies had very small sample sizes (*n* = 5–22), increasing susceptibility to chance findings and sampling error (55).

Given these multiple, overlapping sources of heterogeneity, the pooled effect estimates should be viewed as approximate, hypothesis-generating findings rather than definitive determinations. Subgroup stratification by baseline BP partially explained heterogeneity but did not eliminate it, underscoring the need for standardized research protocols.

Meta-regression indicated age did not significantly moderate BP-lowering effects within the studied range (44–72 years). However, this should be interpreted cautiously given limited statistical power, restricted age range (lacking data on adults <40 and >75 years), and potential collinearity with other covariates (33, 56). Nevertheless, the absence of age moderation within middle-aged and older adults suggests DRT's antihypertensive benefits may not substantially decay with advancing age—an important finding for elderly populations who bear disproportionate hypertension burden and face higher risks of adverse pharmacological events (57).

This study has several important limitations. First, the small total sample (18 studies, 328 participants; individual studies *n* = 5–22) elevates risk of Type II errors and imprecise estimates, particularly for small subgroups (e.g., stage 1 hypertension, k = 4) (55). Second, as discussed, very high heterogeneity (I^2^ = 87%–95%) reflects substantial inconsistency, limiting reliability of pooled estimates. Third, GRADE ratings of “low” (SBP) and “very low” (DBP) indicate limited confidence in effect estimates due to methodological concerns (nearly 80% of studies had “some concerns” for risk of bias) and potential publication bias (DBP: Egger's *p* = 0.017) (58). Fourth, the age range (44–72 years) excludes younger adults and the very elderly, and inadequate reporting of sex, race, and comorbidities limits generalizability. Fifth, reliance on office BP measurements (subject to white-coat effects) and absence of 24-hour ambulatory BP monitoring data limits clinical validity (54). Sixth, lack of vascular function, autonomic, or biomarker assessments precludes elucidation of underlying mechanisms. Finally, short intervention durations (6–24 weeks) with no long-term follow-up limit conclusions about durability of effects.

Based on these findings and limitations, we recommend: (1) conducting high-quality, adequately powered RCTs with rigorous blinding, allocation concealment, and intention-to-treat analysis, particularly for stage 1 hypertension; (2) implementing factorial designs systematically comparing training parameters (intensity, frequency, volume, duration) across BP subgroups; (3) adopting 24-hour ambulatory BP monitoring as a primary outcome to circumvent office measurement limitations; (4) incorporating vascular function metrics (pulse wave velocity, flow-mediated dilation), autonomic markers (heart rate variability, muscle sympathetic nerve activity), and biomarkers (nitric oxide metabolites, inflammatory cytokines) to elucidate mechanisms (59, 60); and (5) conducting long-term interventions (≥12 months) with post-cessation monitoring (6–12 months) to assess durability and detraining effects (64–66).

## Conclusion

This meta-analysis suggests that dynamic resistance training may reduce blood pressure in adults, with the magnitude of reduction potentially varying by baseline blood pressure level. In the included studies, patients with stage 2 hypertension appeared to experience the most pronounced benefits, suggesting that DRT could be considered as a potentially useful non-pharmacological intervention component for this population, though this recommendation should be viewed as provisional. Generally, there appears to be a trend where higher baseline blood pressure is associated with greater antihypertensive benefits. These effects did not show significant moderation by age or medication status within the studied populations. However, given the low-to-very-low certainty of current evidence, very high inter-study heterogeneity, and methodological limitations of included trials, these findings should be interpreted cautiously and require verification through larger, high-quality, adequately powered randomized controlled trials with standardized protocols before definitive clinical practice recommendations can be made.

## Data Availability

The original contributions presented in the study are included in the article/Supplementary Material, further inquiries can be directed to the corresponding author.
