# Supplementary material for: Effects of dynamic resistance training on blood pressure across different baseline levels: a systematic review and meta-analysis
Source: Front Cardiovasc Med. 2026 May 29;13:1843543. doi: 10.3389/fcvm.2026.1843543 (PMC13260143; doi:10.3389/fcvm.2026.1843543)
Supplement: Supplementary file 1 [file Datasheet1.docx]

TableS1.Detailed Search Strategies for Included Databases

| **Database** | **No.** | **Search Strategy** |  |
| --- | --- | --- | --- |
| PubMed | 1 | "Resistance Training"[MeSH Terms] |  |
|  | 2 | "resistance training"[Title/Abstract] OR "resistance exercise*"[Title/Abstract] |  |
|  | 3 | "strength training"[Title/Abstract] OR "strength exercise*"[Title/Abstract] |  |
|  | 4 | "weight training"[Title/Abstract] OR "weight lifting"[Title/Abstract] OR "weightlifting"[Title/Abstract] |  |
|  | 5 | "dynamic resistance"[Title/Abstract] OR "dynamic exercise*"[Title/Abstract] |  |
|  | 6 | "progressive resistance"[Title/Abstract] OR "isotonic exercise*"[Title/Abstract] |  |
|  | 7 | "muscle strengthening"[Title/Abstract] OR "muscular strength"[Title/Abstract] |  |
|  | 8 | "free weight*"[Title/Abstract] OR "dumbbell*"[Title/Abstract] OR "barbell*"[Title/Abstract] |  |
|  | 9 | "elastic band*"[Title/Abstract] OR "resistance band*"[Title/Abstract] OR "elastic resistance"[Title/Abstract] |  |
|  | 10 | "machine-based"[Title/Abstract] OR "weight machine*"[Title/Abstract] |  |
|  | 11 | #1 OR #2 OR #3 OR #4 OR #5 OR #6 OR #7 OR #8 OR #9 OR #10 |  |
|  | 12 | "Blood Pressure"[MeSH Terms] |  |
|  | 13 | "Hypertension"[MeSH Terms] |  |
|  | 14 | "blood pressure"[Title/Abstract] OR "arterial pressure"[Title/Abstract] |  |
|  | 15 | "systolic"[Title/Abstract] OR "diastolic"[Title/Abstract] |  |
|  | 16 | "hypertension"[Title/Abstract] OR "hypertensive"[Title/Abstract] |  |
|  | 17 | "antihypertensive"[Title/Abstract] OR "BP"[Title/Abstract] OR "SBP"[Title/Abstract] OR "DBP"[Title/Abstract] |  |
|  | 18 | #12 OR #13 OR #14 OR #15 OR #16 OR #17 |  |
|  | 19 | "Randomized Controlled Trial"[Publication Type] |  |
|  | 20 | "Randomized Controlled Trials as Topic"[MeSH Terms] |  |
|  | 21 | "randomized"[Title/Abstract] OR "randomised"[Title/Abstract] |  |
|  | 22 | "random allocation"[Title/Abstract] OR "randomly"[Title/Abstract] |  |
|  | 23 | "controlled trial*"[Title/Abstract] OR "clinical trial*"[Title/Abstract] |  |
|  | 24 | "RCT"[Title/Abstract] OR "placebo"[Title/Abstract] |  |
|  | 25 | #19 OR #20 OR #21 OR #22 OR #23 OR #24 |  |
|  | 26 | #11 AND #18 AND #25 |  |
| Embase | 1 | 'resistance training'/exp |  |
|  | 2 | 'resistance training':ti,ab OR 'resistance exercise*':ti,ab |  |
|  | 3 | 'strength training':ti,ab OR 'strength exercise*':ti,ab |  |
|  | 4 | 'weight training':ti,ab OR 'weight lifting':ti,ab OR 'weightlifting':ti,ab |  |
|  | 5 | 'dynamic resistance':ti,ab OR 'dynamic exercise*':ti,ab |  |
|  | 6 | 'progressive resistance':ti,ab OR 'isotonic exercise*':ti,ab |  |
|  | 7 | 'muscle strengthening':ti,ab OR 'muscular strength':ti,ab |  |
|  | 8 | 'free weight*':ti,ab OR 'dumbbell*':ti,ab OR 'barbell*':ti,ab |  |
|  | 9 | 'elastic band*':ti,ab OR 'resistance band*':ti,ab OR 'elastic resistance':ti,ab |  |
|  | 10 | 'machine-based':ti,ab OR 'weight machine*':ti,ab |  |
|  | 11 | #1 OR #2 OR #3 OR #4 OR #5 OR #6 OR #7 OR #8 OR #9 OR #10 |  |
|  | 12 | 'blood pressure'/exp |  |
|  | 13 | 'hypertension'/exp |  |
|  | 14 | 'blood pressure':ti,ab OR 'arterial pressure':ti,ab |  |
|  | 15 | 'systolic':ti,ab OR 'diastolic':ti,ab |  |
|  | 16 | 'hypertension':ti,ab OR 'hypertensive':ti,ab |  |
|  | 17 | 'antihypertensive':ti,ab OR 'BP':ti,ab OR 'SBP':ti,ab OR 'DBP':ti,ab |  |
|  | 18 | #12 OR #13 OR #14 OR #15 OR #16 OR #17 |  |
|  | 19 | 'randomized controlled trial'/exp |  |
|  | 20 | 'randomized':ti,ab OR 'randomised':ti,ab |  |
|  | 21 | 'random allocation':ti,ab OR 'randomly':ti,ab |  |
|  | 22 | 'controlled trial*':ti,ab OR 'clinical trial*':ti,ab |  |
|  | 23 | 'RCT':ti,ab OR 'placebo':ti,ab |  |
|  | 24 | #19 OR #20 OR #21 OR #22 OR #23 |  |
|  | 25 | #11 AND #18 AND #24 |  |
| Cochrane Library | 1 | MeSH descriptor: [Resistance Training] explode all trees |  |
|  | 2 | ("resistance training" OR "resistance exercise*"):ti,ab,kw |  |
|  | 3 | ("strength training" OR "strength exercise*"):ti,ab,kw |  |
|  | 4 | ("weight training" OR "weight lifting" OR "weightlifting"):ti,ab,kw |  |
|  | 5 | ("dynamic resistance" OR "dynamic exercise*"):ti,ab,kw |  |
|  | 6 | ("progressive resistance" OR "isotonic exercise*"):ti,ab,kw |  |
|  | 7 | ("muscle strengthening" OR "free weight*" OR "dumbbell*" OR "barbell*"):ti,ab,kw |  |
|  | 8 | ("elastic band*" OR "resistance band*" OR "elastic resistance"):ti,ab,kw |  |
|  | 9 | #1 OR #2 OR #3 OR #4 OR #5 OR #6 OR #7 OR #8 |  |
|  | 10 | MeSH descriptor: [Blood Pressure] explode all trees |  |
|  | 11 | MeSH descriptor: [Hypertension] explode all trees |  |
|  | 12 | ("blood pressure" OR "arterial pressure"):ti,ab,kw |  |
|  | 13 | ("systolic" OR "diastolic" OR "hypertension" OR "hypertensive"):ti,ab,kw |  |
|  | 14 | ("SBP" OR "DBP" OR "antihypertensive"):ti,ab,kw |  |
|  | 15 | #10 OR #11 OR #12 OR #13 OR #14 |  |
|  | 16 | #9 AND #15 (Filter: Cochrane Central Register of Controlled Trials) | [n] |
| Web of Science | 1 | TS = ("resistance training" OR "resistance exercise*" OR "strength training" OR "strength exercise*") |  |
|  | 2 | TS = ("weight training" OR "weight lifting" OR "weightlifting" OR "dynamic resistance" OR "dynamic exercise*") |  |
|  | 3 | TS = ("progressive resistance" OR "isotonic exercise*" OR "muscle strengthening" OR "muscular strength") |  |
|  | 4 | TS = ("free weight*" OR "dumbbell*" OR "barbell*" OR "elastic band*" OR "resistance band*" OR "elastic resistance" OR "machine-based") |  |
|  | 5 | #1 OR #2 OR #3 OR #4 |  |
|  | 6 | TS = ("blood pressure" OR "arterial pressure" OR "systolic" OR "diastolic") |  |
|  | 7 | TS = ("hypertension" OR "hypertensive" OR "antihypertensive" OR "SBP" OR "DBP") |  |
|  | 8 | #6 OR #7 |  |
|  | 9 | TS = ("randomized" OR "randomised" OR "random allocation" OR "randomly" OR "controlled trial*" OR "clinical trial*" OR "RCT" OR "placebo") |  |
|  | 10 | #5 AND #8 AND #9 (Database: SCI-EXPANDED, SSCI) |  |
| Medline (OVID) | 1 | exp Resistance Training/ |  |
|  | 2 | (resistance training or resistance exercise*).ti,ab. |  |
|  | 3 | (strength training or strength exercise*).ti,ab. |  |
|  | 4 | (weight training or weight lifting or weightlifting).ti,ab. |  |
|  | 5 | (dynamic resistance or dynamic exercise* or progressive resistance).ti,ab. |  |
|  | 6 | (isotonic exercise* or muscle strengthening or muscular strength).ti,ab. |  |
|  | 7 | (free weight* or dumbbell* or barbell* or elastic band* or resistance band* or elastic resistance or machine-based).ti,ab. |  |
|  | 8 | #1 OR #2 OR #3 OR #4 OR #5 OR #6 OR #7 |  |
|  | 9 | exp Blood Pressure/ |  |
|  | 10 | exp Hypertension/ |  |
|  | 11 | (blood pressure or arterial pressure or systolic or diastolic).ti,ab. |  |
|  | 12 | (hypertension or hypertensive or antihypertensive or BP or SBP or DBP).ti,ab. |  |
|  | 13 | #9 OR #10 OR #11 OR #12 |  |
|  | 14 | randomized controlled trial.pt. |  |
|  | 15 | (randomized or randomised or randomly or random allocation).ti,ab. |  |
|  | 16 | (controlled trial* or clinical trial* or RCT or placebo).ti,ab. |  |
|  | 17 | #14 OR #15 OR #16 |  |
|  | 18 | #8 AND #13 AND #17 |  |
| APA PsycNet | 1 | Any Field: "resistance training" OR "resistance exercise" OR "strength training" OR "strength exercise" |  |
|  | 2 | Any Field: "weight training" OR "weight lifting" OR "weightlifting" OR "dynamic resistance" |  |
|  | 3 | Any Field: "progressive resistance" OR "isotonic exercise" OR "muscle strengthening" |  |
|  | 4 | Any Field: "free weight" OR "dumbbell" OR "barbell" OR "elastic band" OR "resistance band" |  |
|  | 5 | #1 OR #2 OR #3 OR #4 |  |
|  | 6 | Any Field: "blood pressure" OR "arterial pressure" OR "systolic" OR "diastolic" |  |
|  | 7 | Any Field: "hypertension" OR "hypertensive" OR "antihypertensive" |  |
|  | 8 | #6 OR #7 |  |
|  | 9 | Any Field: "randomized" OR "randomised" OR "controlled trial" OR "clinical trial" OR "RCT" |  |
|  | 10 | #5 AND #8 AND #9 |  |
| ProQuest | 1 | ti,ab("resistance training" OR "resistance exercise" OR "strength training" OR "strength exercise") |  |
|  | 2 | ti,ab("weight training" OR "weight lifting" OR "weightlifting" OR "dynamic resistance" OR "dynamic exercise") |  |
|  | 3 | ti,ab("progressive resistance" OR "isotonic exercise" OR "muscle strengthening" OR "free weight" OR "dumbbell" OR "barbell" OR "elastic band" OR "resistance band") |  |
|  | 4 | #1 OR #2 OR #3 |  |
|  | 5 | ti,ab("blood pressure" OR "arterial pressure" OR "systolic" OR "diastolic" OR "hypertension" OR "hypertensive" OR "SBP" OR "DBP") |  |
|  | 6 | ti,ab("randomized" OR "randomised" OR "controlled trial" OR "clinical trial" OR "RCT" OR "randomly") |  |
|  | 7 | #4 AND #5 AND #6 (Filter: Peer-reviewed, Scholarly Journals) |  |
| SpringerLink | 1 | ("resistance training" OR "resistance exercise" OR "strength training" OR "weight training" OR "dynamic resistance" OR "progressive resistance") AND ("blood pressure" OR "hypertension" OR "systolic" OR "diastolic") AND ("randomized" OR "randomised" OR "controlled trial" OR "RCT") (Filter: Article type: Research Article; Discipline: Medicine & Public Health) |  |
| Scopus | 1 | TITLE-ABS-KEY("resistance training" OR "resistance exercise*" OR "strength training" OR "strength exercise*") |  |
|  | 2 | TITLE-ABS-KEY("weight training" OR "weight lifting" OR "weightlifting" OR "dynamic resistance" OR "dynamic exercise*") |  |
|  | 3 | TITLE-ABS-KEY("progressive resistance" OR "isotonic exercise*" OR "muscle strengthening" OR "muscular strength") |  |
|  | 4 | TITLE-ABS-KEY("free weight*" OR "dumbbell*" OR "barbell*" OR "elastic band*" OR "resistance band*" OR "elastic resistance" OR "machine-based") |  |
|  | 5 | #1 OR #2 OR #3 OR #4 |  |
|  | 6 | TITLE-ABS-KEY("blood pressure" OR "arterial pressure" OR "systolic" OR "diastolic") |  |
|  | 7 | TITLE-ABS-KEY("hypertension" OR "hypertensive" OR "antihypertensive" OR "SBP" OR "DBP") |  |
|  | 8 | #6 OR #7 |  |
|  | 9 | TITLE-ABS-KEY("randomized" OR "randomised" OR "random allocation" OR "randomly" OR "controlled trial*" OR "clinical trial*" OR "RCT" OR "placebo") |  |
|  | 10 | #5 AND #8 AND #9 (Filter: Document type: Article; Source type: Journal) |  |

TableS2.PICOS-Based Inclusion and Exclusion Criteria for Study Selection

| **PICOS Domain** | **Inclusion Criteria** | **Exclusion Criteria** |
| --- | --- | --- |
| **P (Population)** | ① Adults aged ≥ 18 years, regardless of sex or ethnicity;② Participants with any baseline blood pressure level (including normal BP, high-normal BP, stage 1 hypertension, and stage 2 hypertension), as classified by the 2017 ACC/AHA guidelines or equivalent international guidelines;③ Both medicated (receiving antihypertensive pharmacotherapy) and unmedicated participants were eligible;④ No restrictions on health status (including but not limited to healthy individuals, patients with hypertension, type 2 diabetes, obesity, chronic kidney disease, or other chronic conditions). | ① Participants aged < 18 years (children and adolescents);② Pregnant or postpartum women;③ Participants with acute cardiovascular events (e.g., acute myocardial infarction, acute stroke) within the past 6 months;④ Participants with secondary hypertension (e.g., renal artery stenosis, pheochromocytoma, primary aldosteronism);⑤ Participants with unstable hemodynamic conditions or acute illness. |
| **I (Intervention)** | ① Dynamic resistance training (DRT), defined as exercise involving concentric and/or eccentric muscle contractions through a full range of motion against an external resistance;② Acceptable training modalities included: free weights (dumbbells, barbells, kettlebells), weight machines, elastic bands/tubes, bodyweight exercises, or any combination thereof;③ Structured DRT program with a clearly described protocol (including at least: training frequency, intensity, sets, repetitions, and duration);④ Intervention duration ≥ 4 weeks;⑤ DRT performed as the sole exercise intervention or as the primary component of the intervention. | ① Isometric resistance training (e.g., isometric handgrip, wall sit);② Aerobic exercise alone (e.g., walking, jogging, cycling, swimming);③ Combined aerobic and resistance training (concurrent training) where the effects of DRT could not be isolated;④ Acute (single-bout) resistance exercise studies without a chronic training program;⑤ Whole-body vibration training, electrical muscle stimulation, or other passive modalities;⑥ Yoga, Tai Chi, Pilates, or other mind-body exercises as the sole intervention;⑦ Intervention duration < 4 weeks;⑧ Interventions without a clearly described DRT protocol. |
| **C (Comparison)** | ① Non-exercising control group (e.g., usual care, waitlist control, no intervention, maintaining habitual activities);② Sham/attention control group (e.g., stretching-only, health education sessions) that did not include any structured resistance or aerobic exercise component. | ① Active exercise control group (e.g., aerobic exercise, combined training, alternative resistance training);② Pharmacological intervention as the sole comparator;③ Studies without a control group (single-arm pre-post studies). |
| **O (Outcomes)** | ① Primary outcomes: changes in resting systolic blood pressure (SBP) and/or diastolic blood pressure (DBP) measured in mmHg;② Blood pressure measured at rest using validated and standardized methods (e.g., auscultatory sphygmomanometer, automated oscillometric device, or ambulatory blood pressure monitoring);③ Sufficient data reported to calculate the mean difference (MD) and standard deviation (SD) of BP changes, or data that could be derived from available information (e.g., 95% CI, SE, p-values, or individual data points). | ① Studies reporting only ambulatory blood pressure monitoring (ABPM) results without resting BP values;② Studies reporting only exercise-induced (acute) BP responses without chronic resting BP changes;③ Studies reporting BP outcomes only in graphical format without extractable numerical data;④ Insufficient data to calculate effect sizes, and authors could not be contacted or did not respond. |
| **S (Study Design)** | ① Randomized controlled trials (RCTs), including both parallel-group and crossover designs;② Published in English;③ Published as full-text peer-reviewed journal articles. | ① Non-randomized controlled trials, quasi-experimental studies;② Observational studies (cohort, case-control, cross-sectional);③ Systematic reviews, meta-analyses, narrative reviews, editorials, commentaries, letters, or conference abstracts;④ Case reports or case series;⑤ Animal studies or in vitro studies;⑥ Theses and dissertations, unpublished manuscripts;⑦ Non-English language publications;⑧ Duplicate publications (the most complete or most recent version was retained). |

FigureS1.Baseline Characteristics of Included Studies


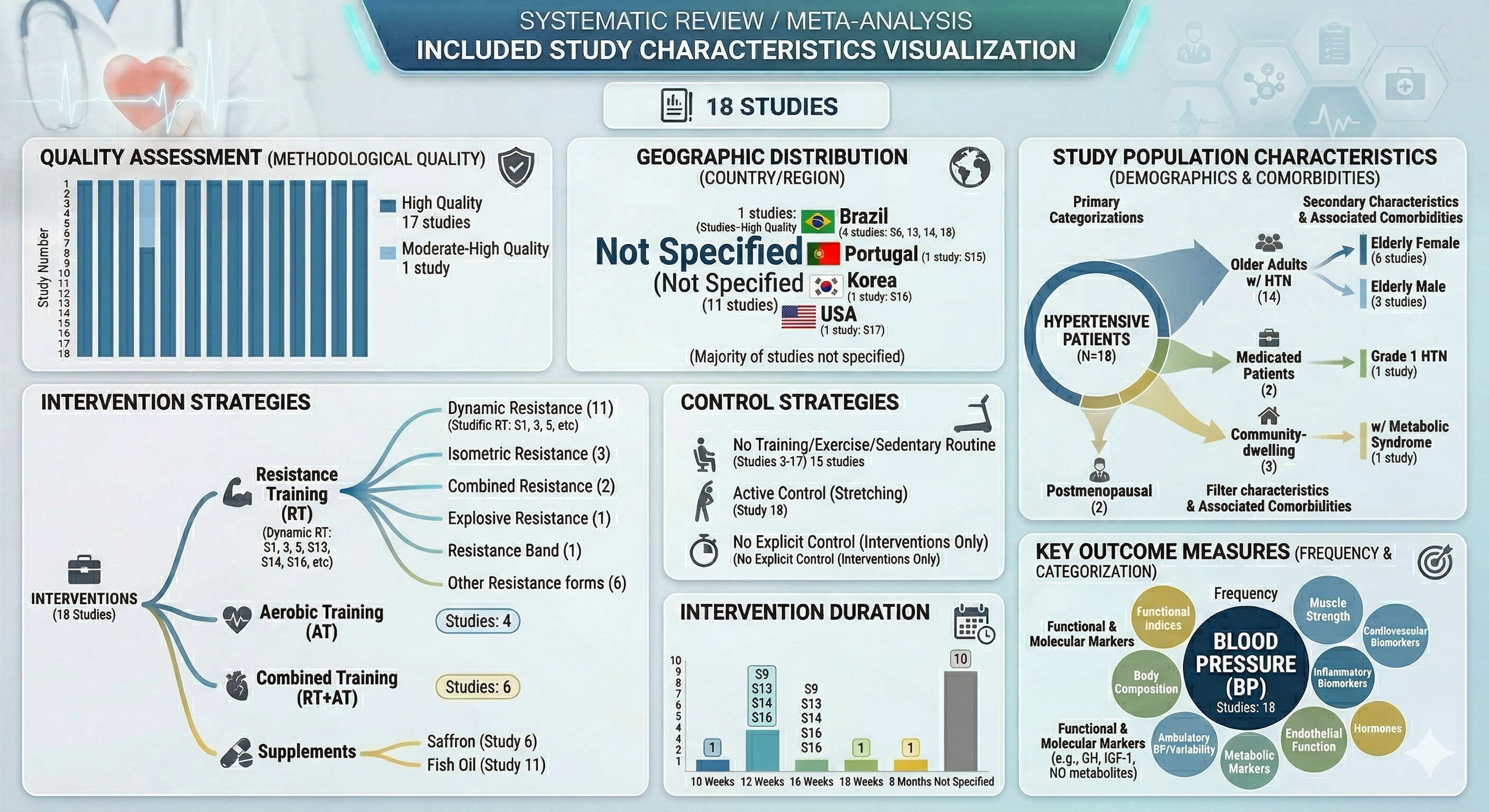


Table S3. GRADE Evidence Profile for the Effects of Dynamic Resistance Training on Blood Pressure

| **Outcome** | **No. of studies (participants)** | **Risk of bias** | **Inconsistency** | **Indirectness** | **Imprecision** | **Publication bias** | **Overall certainty** | **Effect estimate, MD [95% CI]** |
| --- | --- | --- | --- | --- | --- | --- | --- | --- |
| SBP | k = [27] (n = [328]) | ⊕ Serious ^a^ | ⊕ Serious ^b^ | Not serious | Not serious | Not detected ^d^ | ⊕⊕◯◯ LOW | −7.57[−9.40, −5.74] mmHg |
| DBP | k = [26] (n = 313) | ⊕ Serious ^a^ | ⊕ Serious ^c^ | Not serious | Not serious | ⊕ Serious ^e^ | ⊕◯◯◯ VERY LOW | −4.73[−6.41, −3.05] mmHg |

Note：^a^ Downgraded by one level for serious risk of bias: approximately 80% of included studies were rated as having "some concerns" by the Cochrane Risk of Bias 2.0 tool（Rob 2），primarily due to inadequate blinding of participants and outcome assessors.

^b^ Downgraded by one level for serious inconsistency: substantial heterogeneity was observed across studies (I² = 87.0%, P < 0.001).

^c^ Downgraded by one level for serious inconsistency: substantial heterogeneity was observed across studies (I² = 95.1%, P < 0.001).

^d^ Egger's test did not indicate significant publication bias for SBP (P = 0.165 ); visual inspection of the funnel plot revealed approximate symmetry.

^e^ Downgraded by one level for serious suspicion of publication bias: Egger's regression test indicated significant asymmetry (P = 0.017), and visual inspection of the funnel plot revealed potential asymmetry.
